# Local Interactions in Aqueous Ethanol Solution Revealed by the C=O Stretching Probe

**DOI:** 10.3390/molecules30071524

**Published:** 2025-03-29

**Authors:** Zhiqiang Wang, Chi Chen, Ruiting Zhang, Lin Ma, Ke Lin

**Affiliations:** School of Physics, Xidian University, Xi’an 710071, China; zqwang@xidian.edu.cn (Z.W.); cchi479@163.com (C.C.); rtzhang@xidian.edu.cn (R.Z.); linma@xidian.edu.cn (L.M.)

**Keywords:** local interactions, C=O stretching vibration, hydrogen bond, ethyl acetate, ethanol–water, Raman spectrum

## Abstract

Accurately identifying local interactions such as hydrophilicity and hydrophobicity is of critical importance in regulating the functions of amphiphilic biomolecules, but in situ identification methods for such interactions are still lacking. This study proposes a probe based on carbonyl (C=O) stretching vibration to study the hydrophilic and hydrophobic interactions in amphiphilic alcohol–water systems. A combination of theoretical calculations and Raman spectroscopy experiments is employed to investigate the molecular interactions of ethyl acetate C=O in an ethanol aqueous solution, as well as the reasons behind the splitting of spectral peaks. The results indicate that the spectral peak splitting of the C=O stretching vibration is attributed to ethyl acetate existing in different hydrophilic and hydrophobic environments. Specifically, the two low-wavenumber components arise from the formation of double and single hydrogen bonds between C=O and water or ethanol, respectively, while the high-wavenumber component is attributed to the interaction between C=O and the hydrophobic alkyl group. These findings suggest that the C=O stretching vibration of esters is sensitive to the surrounding hydrophilic and hydrophobic environments, thereby indicating its potential as a useful probe for identifying hydrophilic and hydrophobic interactions.

## 1. Introduction

Hydrophilic–hydrophobic interactions play a crucial role in numerous biological, physical, and chemical processes [1,2]. In recent years, the self-assembly of amphiphilic molecules has garnered significant attention due to its extensive applications in materials science, medicine, and gene delivery [3,4,5,6,7,8]. Amphiphilic molecules, which possess both hydrophilic and hydrophobic groups, exhibit self-assembly capabilities. They have been widely utilized to model biological systems, such as the assembly of lipids and proteins, and hold substantial promise in the field of bio-nanotechnology [9,10,11].

Hydrophilic–hydrophobic interactions arise from the mutual attraction or repulsion between the hydrophilic and hydrophobic groups of a molecule and water molecules, leading to the aggregation of hydrophobic groups. Hydrophobic groups, such as alkyl groups, are typically non-polar. It is important to note that hydrophobic interactions are not bonds, but rather non-specific interactions that are stronger than van der Waals dispersion forces [12]. Many biological macromolecules, including proteins and DNA, are generally amphiphilic, containing both hydrophobic and hydrophilic groups. In these molecules, the hydrophobic groups are typically sequestered internally, while the hydrophilic groups are oriented outward. Hydrophobic interactions can induce protein folding and conformational changes, thereby influencing their activity and stability [13,14]. This conformational change is crucial for the regulation of protein function. However, there remains ongoing debate regarding the impact of hydrophobic interactions on the structure of aqueous solutions.

To simplify the research process, the ethanol–water system serves as an ideal model for investigating hydrophilic–hydrophobic interactions. Ethanol is an amphiphilic small molecule that possesses both hydrophobic alkyl groups and hydrophilic hydroxyl groups, making it soluble in water [15,16,17,18]. The hydroxyl groups (O-H) in ethanol form hydrogen bonds with the oxygen atoms in water molecules, demonstrating hydrophilicity. As the ratio of ethanol to water molecules varies, the microstructure of the solution also undergoes changes. Specifically, when the molar content of ethanol reaches approximately 30%, ethanol (ET) transitions from forming 1ET:2H_2_O molecular complexes to 1ET:1H_2_O complexes [19]. Ethanol can aggregate through hydrogen bonding between its O-H groups, which exposes the hydrophobic alkyl groups to the exterior [20,21]. In an ethanol–water solution, a tetrahedral water network is established around the hydrophobic group [22,23]. This cage-like hydration network permits the stable existence of the hydrophobic group in water [18]. When the content of the hydrophobic group increases, the hydration layer network cannot completely cover the exposed hydrophobic group. Furthermore, the interactions between water molecules and alcohol molecules also influence the configuration of the alcohol molecules [24]. Hydrogen bonds are widely present in solvents such as water and alcohols, which can affect molecular structure and dynamic behavior, as well as having a significant impact on hydrophilicity and hydrophobicity [25,26,27,28]. In the study of solute solvent interactions, the characteristics of hydrogen bonds are typically detected through vibrational spectroscopic techniques such as infrared spectroscopy (IR), Raman spectroscopy, vibrational circular dichroism spectroscopy (VCD), and Raman optical activity (ROA), which can provide detailed information about molecular conformation and hydrogen bond directionality [29,30,31]. Therefore, the microstructure in the ethanol–water binary solution is closely related to the hydrophilic and hydrophobic environment.

To date, hydrophobic and hydrophilic phenomena have primarily been investigated by observing changes in the O-H stretching vibration band of water [9,32,33,34]. However, a recent study has proposed that the stretching vibration frequency of carbonyl groups (C=O) in esters is sensitive to its environment [35]. Ethyl acetate (EA), a relatively common small molecule ester containing a C=O group, exhibits a broad C=O vibration spectrum when dissolved in water. In contrast, this spectrum splits when the solvent is alcohol. Significant research has been conducted to elucidate the spectral splitting phenomenon of C=O in proton-donating solvents, attributing it to the formation of hydrogen bonds between molecules [35,36]. However, the explanation based solely on hydrogen bond formation is insufficient, as both water and alcohol are capable of forming hydrogen bonds with C=O.

To elucidate the phenomenon of spectral peak splitting, this study conducted a comprehensive investigation into the intermolecular interactions of the C=O group in ethyl acetate within an ethanol–water binary system, utilizing both theoretical calculations and Raman spectroscopy methods. The results indicate that the spectral peak splitting of the C=O stretching vibration is attributed to ethyl acetate existing in varying hydrophilic and hydrophobic environments. These findings suggest that the C=O stretching vibration of esters is sensitive to the surrounding hydrophilic and hydrophobic contexts, and it is anticipated that this sensitivity will serve as a valuable probe for identifying hydrophilic and hydrophobic interactions.

## 2. Results and Discussion

**Figure 1:**
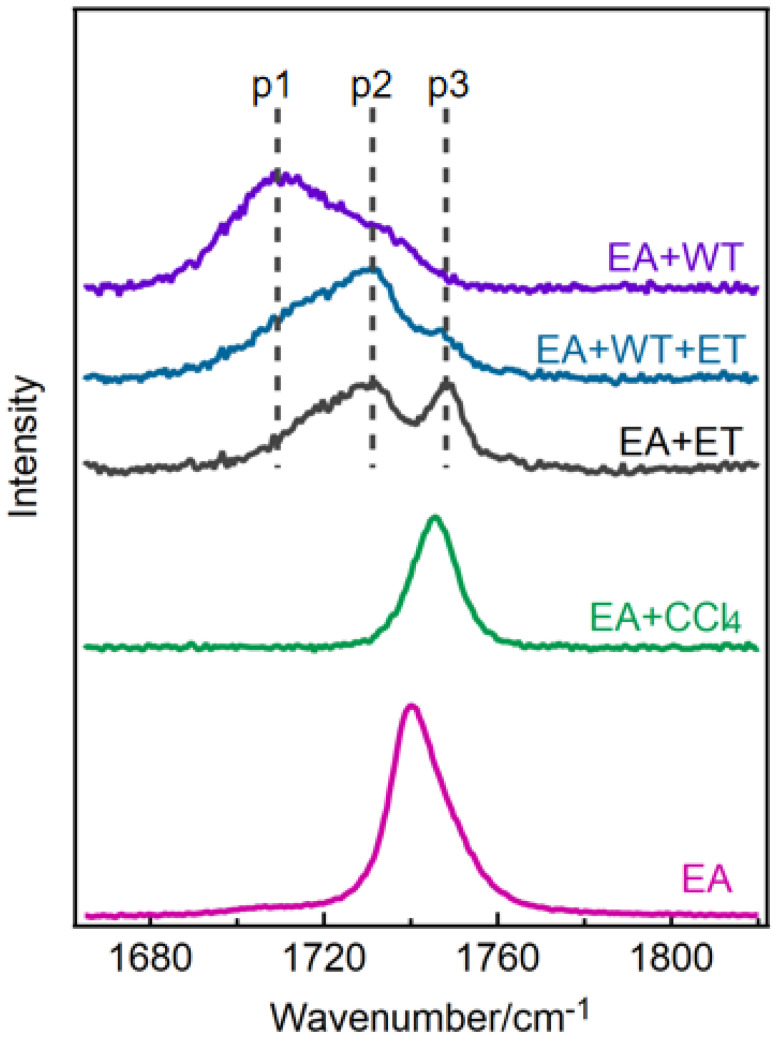
The Raman spectra of C=O stretching vibration of ethyl acetate (EA) and its mixed solutions in carbon tetrachloride (CCl_4_), water (WT), ethanol (ET) and ethanol−water. The molar content of ethyl acetate solution is 1%.

The Raman spectra of ethyl acetate (EA) and its mixed solutions in water (WT), ethanol (ET), and ethanol–water were acquired by analyzing the C=O stretching vibration band. As shown in Figure 1, the C=O stretching vibration peak of pure ethyl acetate (EA) is observed at 1740 cm^−1^, consistent with the reference [37]. The C=O peak of ethyl acetate in water broadens and exhibits a pronounced shoulder peak. Conversely, in ethanol, the peak is segmented into roughly three components, labeled p1, p2, and p3. This splitting has been ascribed to the establishment of hydrogen bonds between the hydroxyl group (O-H) in the ethanol or water molecule and the C=O of ethyl acetate [35,36]. The C=O stretching vibration of ethyl acetate in carbon tetrachloride (CCl_4_) displays a symmetrical peak (Figure 1). This phenomenon arises because carbon tetrachloride is incapable of forming hydrogen bonds with the C=O of ethyl acetate, resulting in the lack of peak splitting.

Despite the ability of water and ethanol to form hydrogen bonds with the C=O of ethyl acetate, the behavior of the C=O stretching vibration peak exhibits notable differences. In water, the peak is observed to split into two components (p1, p2), while in ethanol, it splits into three components (p1, p2, p3). When in an ethanol–water solution, the peak again splits into three components (p1, p2, p3), although the intensity of p3 is diminished compared to that observed in pure ethanol (Figure 1). To explore the reason for the splitting of the C=O stretching vibration peak, we conducted the following series of experiments and calculations.

### 2.1. The Effect of Different Ethyl Acetate Conformers

**Figure 2:**
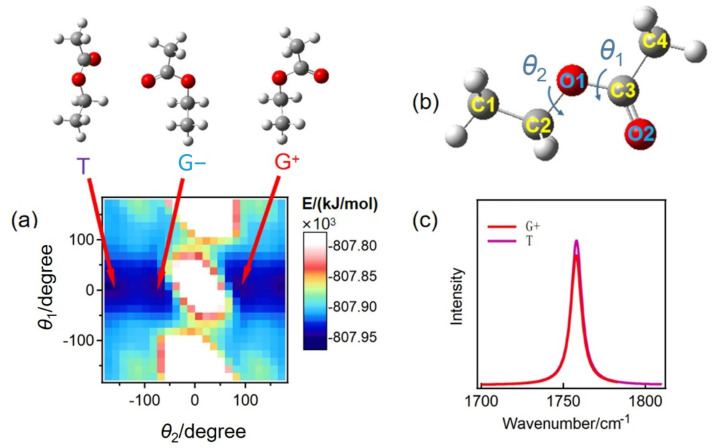
(**a**) The scanned potential energy surface of the ethyl acetate molecule. (**b**) The molecular structure of ethyl acetate contains two variable dihedral angles. (**c**) Calculated vibration frequencies of C=O in three stable conformers of ethyl acetate.

A chemical bond may exhibit varying vibration frequencies across different conformers [38]. To investigate the influence of ethyl acetate conformers on peak splitting, the potential energy surface of the ethyl acetate molecule was analyzed using the B3LYP/6–311++G** basis set (Figure 2a). Given the symmetrical distribution of the methyl groups, their rotation does not lead to the formation of additional conformers of the ethyl acetate molecule. Consequently, it is sufficient to rotate the two single bonds of the ethyl acetate molecule (Figure 2b) to compute its molecular potential energy at various dihedral angles.

As shown in Figure 2b, *θ*_1_ represents the dihedral angle formed by the atoms C2O1-C3O2 and *θ*_2_ represents the dihedral angle formed by the atoms C1C2-O1C3. The scanning range for the two angles extends from −180° to 180°. When the dihedral angles are set to *θ*_1_ = 0° and *θ*_2_ = ±180°, or *θ*_1_ = 0° and *θ*_2_ = ±90°, the molecular potential energy reaches a minimum, indicating that the corresponding molecular configurations are relatively stable. Since *θ*_2_ = +180° and −180° are equivalent angles, three stable conformers were selected for further analysis: *θ*_1_ = 0° and *θ*_2_= −180°, *θ*_1_ = 0° and *θ*_2_ = +90° and *θ*_1_ = 0° and *θ*_2_ = −90°. The spectra of these conformers were calculated and labeled T, G+, and G−. The C=O vibration frequencies of the three conformers of ethyl acetate are nearly identical, situated at 1758 cm^−1^ (Figure 2c). Therefore, the splitting of the C=O vibration spectrum peak observed in Figure 1 cannot be attributed to the formation of conformers.

The presence of aggregates in the ethyl acetate solution may also result in peak splitting due to the non-coincidence effect. However, the depolarization ratio obtained through polarized Raman spectroscopy (Figure S2) suggests that this is not the cause of the observed peak splitting. For more detailed results, please refer to the support information (SI).

### 2.2. The Effect of Hydrophobic Alkyl Groups of Ethanol

Considering the differences in the molecular structures of ethanol and water, the additional component p3 of the C=O stretching vibration peak of ethyl acetate may be attributed to the hydrophobic alkyl groups contained in ethanol molecules. The C=O vibration frequency of ethyl acetate may have a certain shift in different solvents [39,40]. When ethyl acetate is in a hydrophobic alkane solvent, the C=O vibrational frequency is approximately aligned with that of the component p3. For instance, the C=O peak is observed at 1749.6 cm^−1^ in cyclohexane, 1750.5 cm^−1^ in isooctane, and 1748 cm^−1^ in ethanol. Therefore, the component p3 may result from the partial C=O groups of ethyl acetate being in the hydrophobic alkyl environment of ethanol.

**Figure 3:**
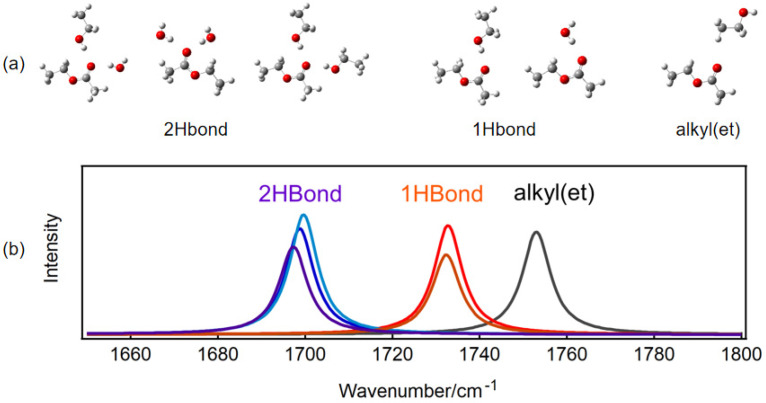
(**a**) The theoretically optimized C=O bond of ethyl acetate forms one hydrogen bond (1HBond), two hydrogen bonds (2HBond), or a structure around the alkyl group of ethanol. The red spheres are oxygen atoms, and the more stable T configuration of ethyl acetate molecule was used here. (**b**) The calculated vibration frequencies of ethyl acetate C=O in the corresponding structure of (**a**).

We optimized the cluster structures formed by ethyl acetate with hydrophilic and hydrophobic groups and calculated the corresponding C=O vibrational frequencies, utilizing the T-configuration of ethyl acetate. As shown in Figure 3a, the result is an optimized stable configuration, where 2Hbond indicates that there are two hydrogen bonds around a C=O bond formed by one ethyl acetate and two water molecules, two ethanol molecules, or one ethanol molecule and one water molecule; 1Hbond indicates that there is one hydrogen bond around a C=O bond, formed by one ethyl acetate molecule and one water molecule or one ethanol molecule; alkyl(et) indicates that the C=O bond is around the hydrophobic alkyl group of ethanol. Figure 3b presents the C=O stretching vibration spectrum calculated based on the corresponding stable structure. The figure illustrates that the vibration frequency of C=O is red-shifted following the formation of hydrogen bonds, with a greater number of hydrogen bonds correlating to a more significant frequency red-shift. This phenomenon occurs because the formation of hydrogen bonds enhances the electron density of the oxygen atom in the C=O bond, leading to a more uniform overall electron density distribution within the C=O bond. The calculated results align well with the experimental findings. The absence of the low-wavenumber component in the C=O peak of ethyl acetate in carbon tetrachloride is attributed to the lack of hydrogen bonds (Figure 1). The calculated C=O vibration frequency of ethyl acetate in an alkyl environment (1753 cm^−1^) shows a slight red-shift compared to the C=O frequency of a single ethyl acetate molecule (1758 cm^−1^, Figure 2c), indicating a relatively weak interaction between C=O and the alkyl groups. However, this interaction is significantly weaker than that observed in hydrogen bonding. The calculated C=O stretching vibration frequency in the presence of the alkyl group closely aligns with the high-wavenumber component p3 of the experimental Raman spectrum, suggesting that the peak splitting observed at 1748 cm^−1^ in the experimental results is a consequence of the hydrophobic effect of the alkyl group.

Based on the experimental and calculation results, it is evident that the C=O stretching vibration spectrum of ethyl acetate in a mixed solution of ethanol and water can be divided into three components (p1, p2, p3). Although the effects of water and ethanol molecules on the positions of components p1 and p2 should be different, theoretical calculations show that the difference in peak positions caused by hydrogen bonds with water or ethanol is very small, so we uniformly use one component, either p1 or p2, to represent two hydrogen bonds or one hydrogen bond. These components can be classified as follows: the low-wavenumber components, p1 and p2, correspond to the oxygen atom of C=O to form two hydrogen bonds and one hydrogen bond with the O-H groups of water or ethanol molecules, respectively, and the high-wavenumber component, p3, is associated with the C=O group in the hydrophobic environment created by the ethanol alkyl group.

**Figure 4:**
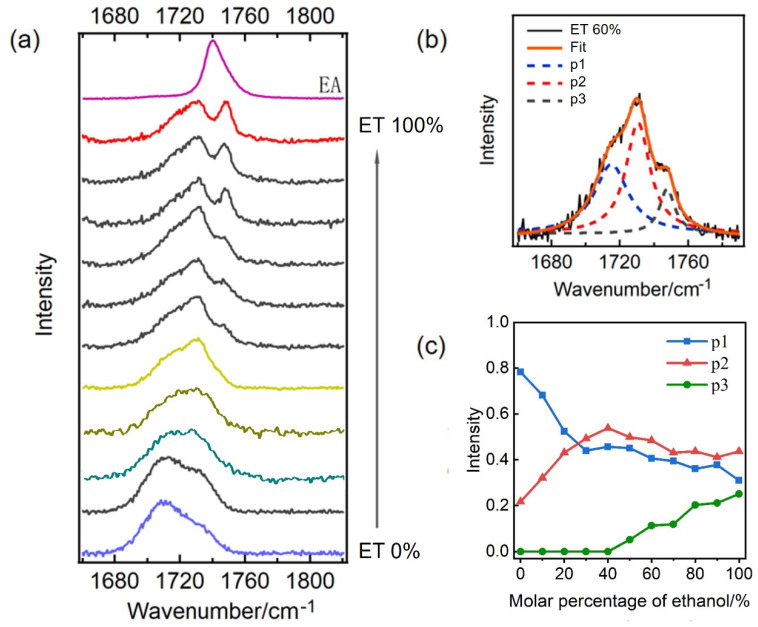
(**a**) The stretching vibration Raman spectra of C=O of ethyl acetate in ethanol–water mixed solutions with different molar ratios. The molar percentage of ethyl acetate is 1%. ET 0% and ET 100%, respectively, represent the ethyl acetate in pure water and pure ethanol, and EA is the pure ethyl acetate. (**b**) Schematic diagram of fitting a spectrum line in Figure 4a with the three components p1, p2, and p3; ET 60% is the selected original spectrum and Fit is the fitted spectrum. (**c**) The ratio of the fitting area of the three components p1 (blue line), p2 (red line), and p3 (black line) to the total spectral peak area changes with the ethanol content.

To further verify our results, we measured the stretching vibration Raman spectra of the C=O bond in ethyl acetate within ethanol–water mixed solutions at varying molar ratios. (Figure 4a). We maintained a molar percentage of ethyl acetate at 1% to ensure effective dispersion in the solution. In Figure 4a, it is evident that the Raman spectrum of the C=O bond exhibits significant changes with varying ethanol content. The labels ET 0% and ET 100% represent ethyl acetate in pure water and pure ethanol, respectively, and EA denotes pure ethyl acetate. At low ethanol concentrations, the spectrum displays a broad band shape, whereas at high ethanol concentrations, notable peak splitting occurs. Furthermore, as the ethanol content increases, the spectral intensity of the p3 component becomes more pronounced.

To enhance our understanding of how these three components vary, we used 3 Gaussian functions to fit the original spectrum shown in Figure 4a. The fitting result for one of the spectrum lines is presented in Figure 4b, demonstrating a strong consistency with the original spectrum. By applying the same fitting method to each spectral line in Figure 4a, we can derive the intensity changes of each component as a function of ethanol content, as illustrated in Figure 4c, with a fitting error of approximately 20%. Notably, Figure 4c indicates that an ethanol content of approximately 30% serves as the inflection point, where the intensities of components p1 and p2 experience significant changes. Specifically, prior to reaching approximately 30% ethanol content, the intensity of p1 decreases rapidly while the intensity of p2 increases sharply, indicating the transformation of double hydrogen bonds into single hydrogen bonds. Following this, after approximately 40% ethanol content, the intensities of both p1 and p2 components gradually decline. Furthermore, the intensity of the hydrophobic component p3 remains at nearly zero when ethanol content is below approximately 40% but begins to increase gradually as the ethanol content exceeds this threshold.

The peak intensity is correlated with the number of molecules in the corresponding structure. From the results presented, it can be concluded that the C=O bond of ethyl acetate can form either single or double hydrogen bonds with water or ethanol molecules. The probability of forming double hydrogen bonds in water is higher, while it is relatively lower in ethanol, which may be because the steric hindrance of forming double hydrogen bonds with ethanol molecules is larger or the energy is higher and unstable. It is also possible that a water molecule and an ethanol molecule may form double hydrogen bonds with the C=O bond when ethanol and water molecules are present simultaneously. When the ethanol content is below 40%, the p3 component approaches zero. This phenomenon occurs because, in the presence of a large number of water molecules, these molecules can create a cage-like structure around the ethanol molecules [16,17]. This configuration effectively encloses the hydrophobic alkyl groups of the ethanol, thereby hindering direct interaction between the carbonyl groups and the hydrophobic alkyl groups. We hope to combine some new higher-level theoretical methods in the future to better explain these experimental phenomena.

Our findings show that the C=O stretching vibration of the ester is sensitive to the surrounding hydrophilic and hydrophobic environment. This sensitivity is promising as a hydrophobic probe to study the interactions of biological molecules, such as nucleic acid–ligand interactions and protein–protein interactions, and can also be used in protein structure and function analysis, such as studying protein folding state and determining the active sites of proteins, as well as in analysis of the surface properties of polymer materials.

## 3. Methods

### 3.1. Experimental Sections

Anhydrous ethanol, ethyl acetate, and carbon tetrachloride were purchased from Sinopharm Chemical Reagent Co., Ltd. (Shanghai, China) with an analytical purity exceeding 99%. All solutions were prepared using distilled water purified through a Millipore Milli-Q system. The preparation process for the ethanol–water mixed solution, with a molar ratio of ethyl acetate at 1%, was as follows: Firstly, 11 portions of ethanol–water mixed solutions with varying molar ratios (ranging from 0% to 100%) were prepared, each with a content of 0.1 mol. Subsequently, 0.001 mol of ethyl acetate was added to each solution. The experimental samples were prepared by weighing at room temperature. After thorough mixing, the samples were placed in a quartz cuvette for Raman spectral collection. The equipment utilized was a backscattering Raman spectroscopy system developed in our laboratory, operating with a laser wavelength of 532 nm and a power output of 900 mW. A detailed description of the experimental setup of the Raman spectrometer (Beijing Zhuoli Hanguang Co., LTD, Beijing, China) (Figure S1) used in this study is also provided in the supporting information (SI).

### 3.2. Computational Details

The geometries, energies, and vibrational frequencies of the ethyl acetate conformers were calculated using the density functional theory (DFT) method. The hybrid functional, which combines Becke’s nonlocal three-parameter exchange with the Lee–Yang–Parr correlation functional (B3LYP), was employed for these calculations [41,42,43]. For molecular orbital expansion, the standard 6–311++G** basis set was utilized in all computations. The vibrational frequencies and intensities were determined using the Gaussian 09W package. What we optimized and calculated was a single ethyl acetate molecule or clusters of ethyl acetate and water or ethanol, without any restrictions added during the optimization process.

## 4. Conclusions

In this study, we conducted a comprehensive investigation into the intermolecular interactions of ethyl acetate within an ethanol–water binary system, focusing particularly on the mechanisms underlying the observed splitting of the C=O stretching vibration peak. This was achieved through a combination of theoretical calculations and Raman spectroscopy experiments. Our findings indicate that the spectral peak splitting of the C=O stretching vibration is attributed to the C=O group existing in different hydrophilic and hydrophobic environments. The two low-wavenumber components, p1 and p2, arise from the formation of double and single hydrogen bonds between C=O and water or ethanol, respectively, while the high-wavenumber component, p3, results from the interaction between C=O and the hydrophobic alkyl group. When the ethanol content is relatively low compared to that of water, the C=O group readily forms one or even two hydrogen bonds with O-H. Conversely, when the ethanol content is high, the proportion of hydrophobic alkyl groups also increases, leading to a greater likelihood that the C=O of the ethyl acetate molecule resides in a hydrophobic environment, thereby increasing the intensity of the high-wavenumber component p3. This work demonstrates that the C=O stretching vibration of esters is sensitive to the surrounding hydrophilic and hydrophobic environments, suggesting its potential as a useful probe for identifying hydrophilic and hydrophobic interactions.

## Data Availability

Data are available upon request from the corresponding author.

## Supplementary Materials

The following supporting information can be downloaded at: https://www.mdpi.com/article/10.3390/molecules30071524/s1. Figure S1: Schematic diagram of the optical path of a Raman spectrometer; Figure S2: The polarized Raman spectrum of ethyl acetate (2% by mole) in ethanol. $I_{⊥}$ and $I_{∥}$ represent the Raman spectrum intensity obtained when it is perpendicular or parallel to the polarization direction of the incident light, respectively. The small circle is the depolarization ratio, and the y-axis on the right represents the magnitude of its value. Refs. [44,45,46] are cited in Supplementary Materials.
