# Supplementary material for: Local Interactions in Aqueous Ethanol Solution Revealed by the C=O Stretching Probe
Source: Molecules. 2025 Mar 29;30(7):1524. doi: 10.3390/molecules30071524 (PMC11990396; doi:10.3390/molecules30071524)
Supplement: Supplementary file 1 [file molecules-30-01524-s001.zip › molecules-3494735-supplementary.pdf]

Supporting Information for

Local Interactions in Aqueous Ethanol Solution Revealed by the C=O Stretching Probe

Zhiqiang Wang, Chi Chen, Ruiting Zhang, Lin Ma and Ke Lin *

School of Physics, Xidian University, Xi'an 710071, China; zqwang@xidian.edu.cn (Z.W.); cchi479@163.com (C.C.); rtzhang@xidian.edu.cn (R.Z.); linma@xidian.edu.cn (L.M.)

* Correspondence: klin@xidian.edu.cn; Tel.: +86-29-88202558

1. Raman spectrometer

The experiments used a self-built Raman spectrometer equipped with a 532 nm continuous laser (Coherent, GenesisMX532 - 1000), a triple monochromator (Beijing Zhuoli Hanguang Instrument Co., Ltd., Omni λ -180D and Omniλ-5008i) and an electrically cooled CCD (Andor, DR-316B-LDC-DD), with a spectral resolution of ~2.0 cm-1. The sample was excited and the Raman spectra were collected using backscattering geometry.

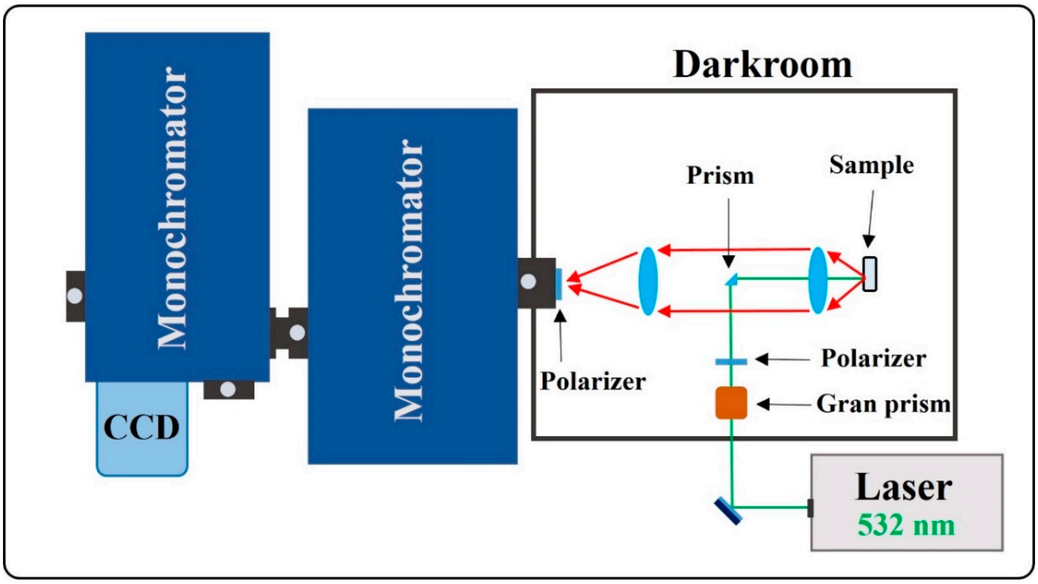

Figure S1. Schematic diagram of the optical path of a Raman spectrometer.

2. The non-coincidence effect of ethyl acetate aggregates

If ethyl acetate aggregates exist in the ethyl acetate solution, the peak splitting may also come from the non-coincidence effect of the aggregates [44-45]. For example, the vibration coupling of molecules will produce peak splitting, which can be distinguished by polarized Raman spectroscopy as symmetric vibration or antisymmetric vibration. Linear polarized light has different polarization degrees for molecules with different vibration directions, so the depolarization ratio ρ can be used to characterize the vibration mode of the molecules [46].

$$\rho = \frac{I_{\perp}}{I_{//}} \quad (1)$$

Where $I_{\perp}$ and $I_{//}$ represent the Raman spectrum intensity obtained when it is perpendicular or parallel to the polarization direction of the incident light, respectively. For a completely symmetrical vibration mode, the polarization direction of the scattered light will be very consistent with that of the incident light, so the value of ρ will be very small. On the contrary, if the molecular vibration is asymmetric, there will be a large difference between the polarization direction of the scattered light and that of the incident light, and the value of ρ will be larger. Usually in polarized Raman spectroscopy, when $\rho < 3/4$, it is called a polarization band, and the corresponding chemical bond is a symmetric vibration mode. When $\rho \geq 3/4$, it is called a depolarization band, and the corresponding chemical bond is an asymmetric vibration mode.

The two C=O couplings of ethyl acetate aggregates can also produce symmetric stretching vibrations and antisymmetric stretching vibrations, which may result in peak splitting in the spectrum. Therefore, we measured the polarized Raman spectrum of ethyl acetate (2% by mole) in ethanol, as shown in Figure S2. The black solid line is the Raman spectrum in the direction parallel to the incident light, and the blue solid line is the Raman spectrum in the direction perpendicular to the incident light. The small red circle is the depolarization ratio, and the y-axis on the right represents the magnitude of its value.

It can be seen that around 1710 ~1758 cm^{-1}, the depolarization ratio is about 0.2, indicating that the C=O vibration is isotropic at this time. According to calculations, if aggregates exist, there will be a component (symmetric mode) with a depolarization ratio of about 0.2 and a component (antisymmetric mode) with a depolarization ratio of about 0.6. Therefore, it can be inferred that the peak splitting is not caused by the non-uniform effect of the aggregate molecules. In addition, the ethyl acetate content is very small, and there are no groups between ethyl acetate molecules that can produce strong interactions to aggregate them together, so under strong dilution, it is unlikely that there will be aggregates between ethyl acetate molecules. Therefore, this three spectral

components p1, p2, and p3 are not caused by ethyl acetate aggregation.

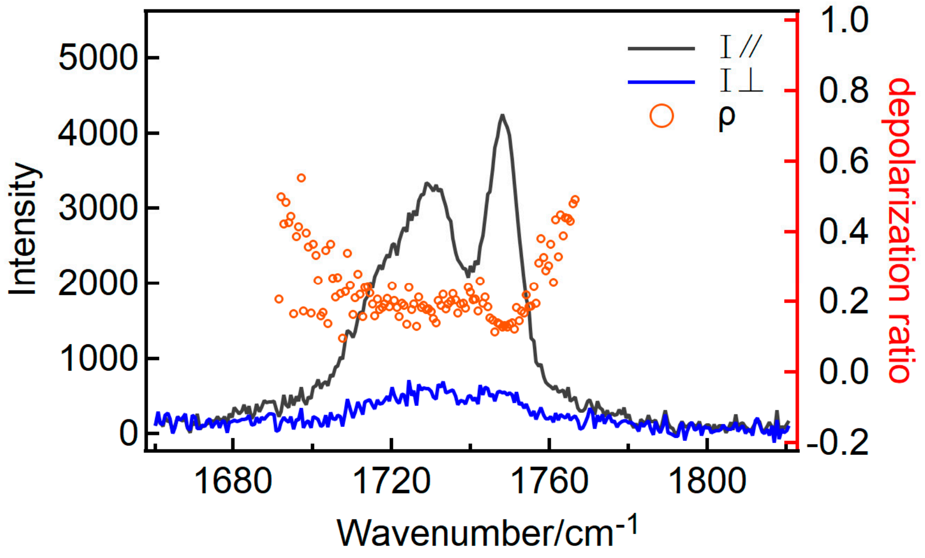

Figure S2. The polarized Raman spectrum of ethyl acetate (2% by mole) in ethanol. $I_{\perp}$ and $I_{//}$ represent the Raman spectrum intensity obtained when it is perpendicular or parallel to the polarization direction of the incident light, respectively. The small circle is the depolarization ratio, and the y-axis on the right represents the magnitude of its value.

References

44. Musso, M.; Giorgini, M. G.; Asenbaum, G. D.; A. The non-coincidence effect in highly diluted acetone-CCl I. Experimental results and theoretical predictions 4 binary mixtures. *Mol. Phys.* **2010,** *92* (1), 97-104.

45. Tukhvatullin, F. H.; Andrews, D. L.; Jumabaev, A.; Tashkenbaev, U. N.; Hushvaktov, H. A.; Absanov, A. A. Polarized components of C=O vibrations Raman spectra for ethylacetate, acetone, and aggregation of molecules. In *International Symposium on Optical Science and Technology*, Proceedings of SPIE: 2002; Vol. 4812, pp 132-138.

46. Murphy, W. F. The Rayleigh depolarization ratio and rotational Raman spectrum of water vapor and the polarizability components for the water molecule. *The Journal of Chemical Physics* **1977,** *67* (12), 5877-5882.
